# Supplementary material for: Ultralong UV/mechano-excited room temperature phosphorescence from purely organic cluster excitons
Source: Nat Commun. 2019 Nov 14;10:5161. doi: 10.1038/s41467-019-13048-x (PMC6856348; doi:10.1038/s41467-019-13048-x)
Supplement: Supplementary file 1 — Supplementary Information [file 41467_2019_13048_MOESM1_ESM.pdf]

Supplementary Information:

Ultralong UV/Mechano-Excited Room Temperature Phosphorescence from Purely Organic Cluster Excitons

Zhang et al.

Supplementary Methods

The initial states of the host and guest molecules are both crystalline after they were purified by silica gel column chromatography and subsequent recrystallization (3×). Take NA/PCP for example, to make the solid-state solution as homogeneous as possible, before melt-casting, the crystals of NA and PCP (wt_{NA}:wt_{PCP} = 1:100) were first ground together in an agate mortar into a fine powdery mixture. As shown in the following photos, the powder was then transferred onto a quartz slide which was the placed on a hot plate stirrer for heating (T = 130 °C). Upon the melting of the host (T_m = 126°C), a colorless and clear solution to the naked eye formed (at a relatively high temperature, the liquid/melted host should have served as a good solvent to dissolve the significantly less amount of NA). Then the hot quartz slide was placed on a test bench at room temperature. Once the melt was cooled to a temperature lower than the T_m of PCP, a white solid quickly formed. The larger pieces of the white solid (not transparent to naked eyes) were then used for PL and ML measurements."

The samples for fs-TA measurements were also prepared via melt-casting similar to the above description. The difference is that the sample for fs-TA measurements was made into largely transparent thin films, since fs-TA measurements require decent transparency. Specifically, a tiny amount (~1 mg) of NA/PCP powder sample was placed in between two quartz slides (3 cm × 3 cm) which was then placed on a hot plate stirrer (T = 130 °C) to melt. Shortly after the hot quartz slides were placed on the test bench at room temperature, the liquid sample quickly became transparent film-like solid in between the two quartz slides, which were later used for fs-TA measurements.

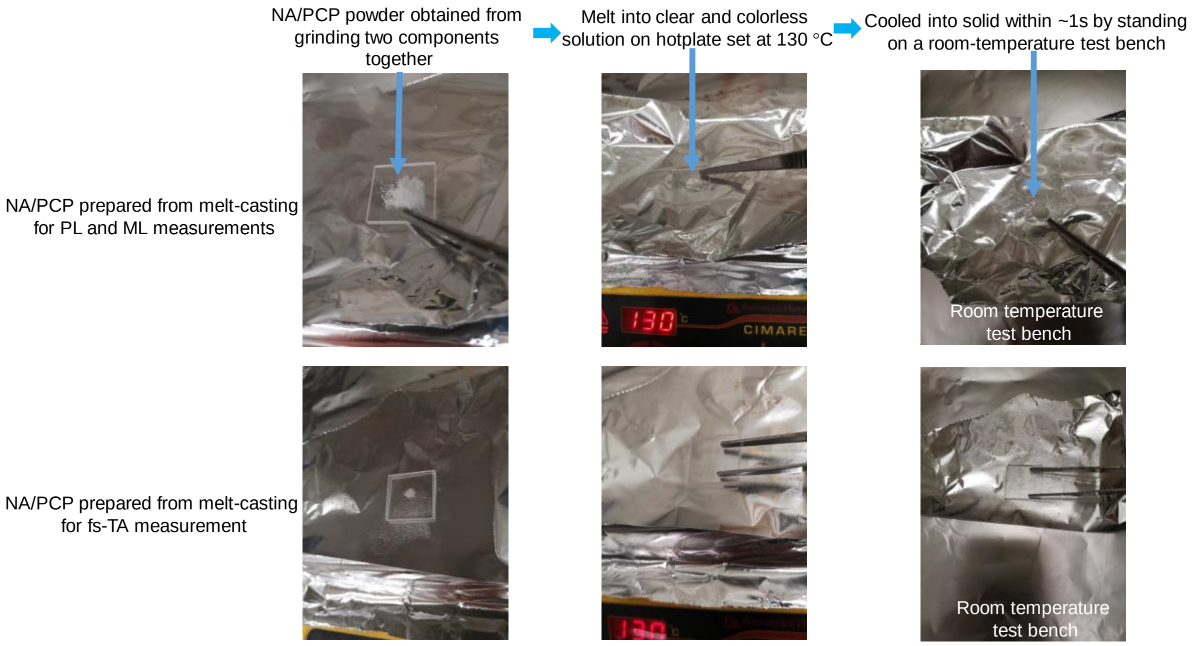

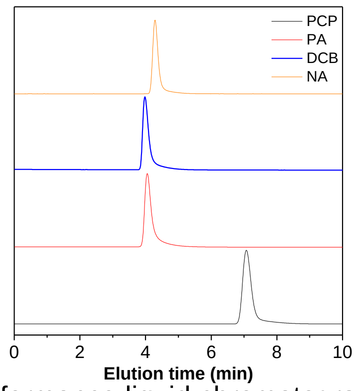

Supplementary Figure 1. High-performance liquid chromatogram spectra of PCP, PA, DCB and NA in acetonitrile solution (50 μM).

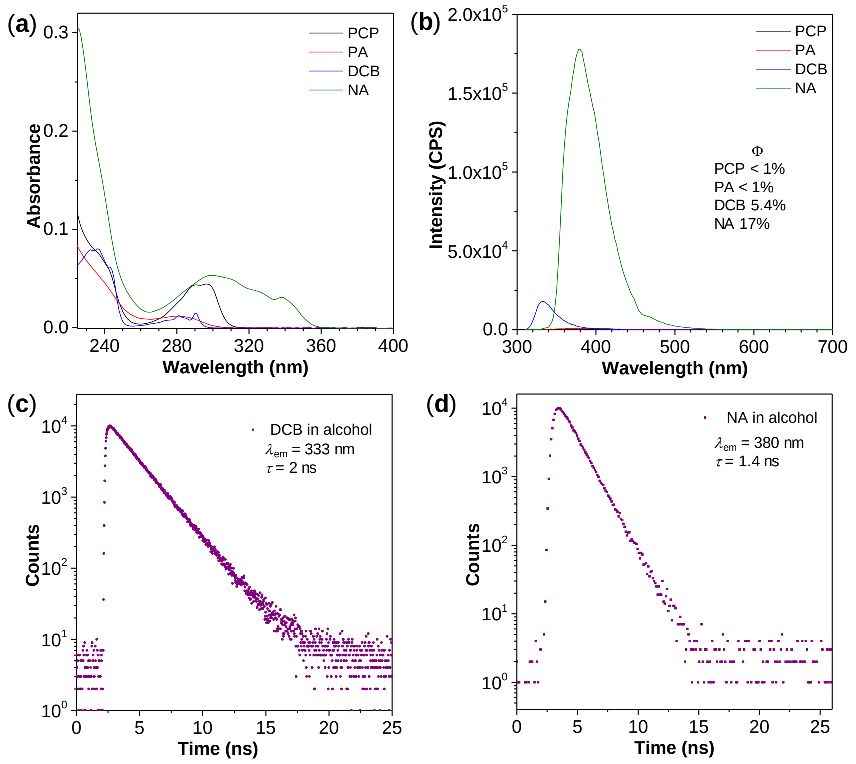

Supplementary Figure 2. (a) UV absorption spectra of PCP, PA, DCB and NA in EtOH/MeOH (*v*/*v* = 4/1, 10^{-5} M) at 298 K. (b) PL spectra of PCP, PA, DCB and NA in EtOH/MeOH (*v*/*v* = 4/1, 10^{-5} M) at 298 K (λ_{ex} = 254 nm). (c) Time-resolved fluorescence decay curve of DCB at 333 nm in EtOH/MeOH (*v*/*v* = 4/1, 10^{-5} M) at 298 K (λ_{ex} = 280 nm). (d) Time-resolved fluorescence decay curve of NA at 380 nm in EtOH/MeOH (*v*/*v* = 4/1, 10^{-5} M) at 298 K (λ_{ex} = 280 nm).

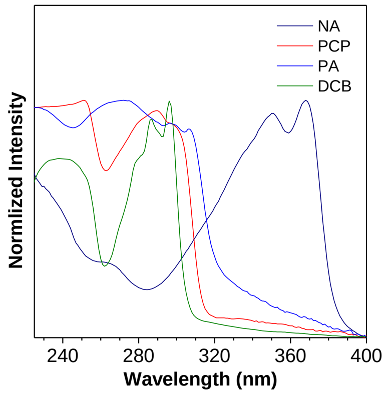

Supplementary Figure 3. UV absorption spectra of PCP, PA, DCB and NA film at 298 K.

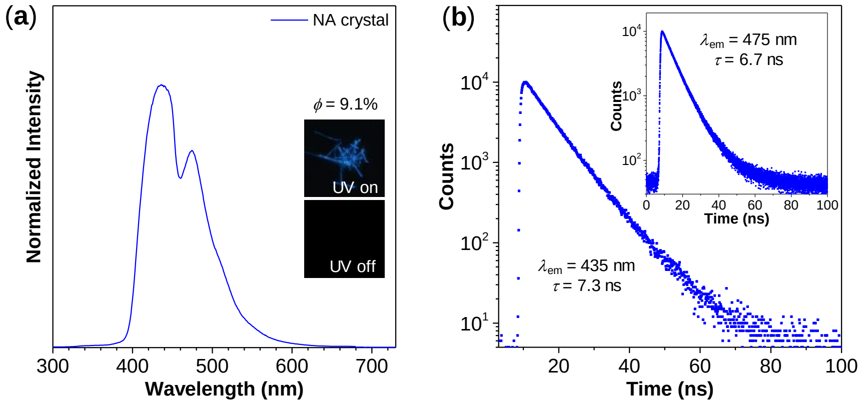

Supplementary Figure 4. (a) Steady-state PL spectrum of NA crystal (λ_{ex} = 254 nm) at 298 K. (b) Time-resolved PL-decay curve of NA crystal at 435 nm and 475 nm (λ_{ex} = 280 nm) at 298 K.

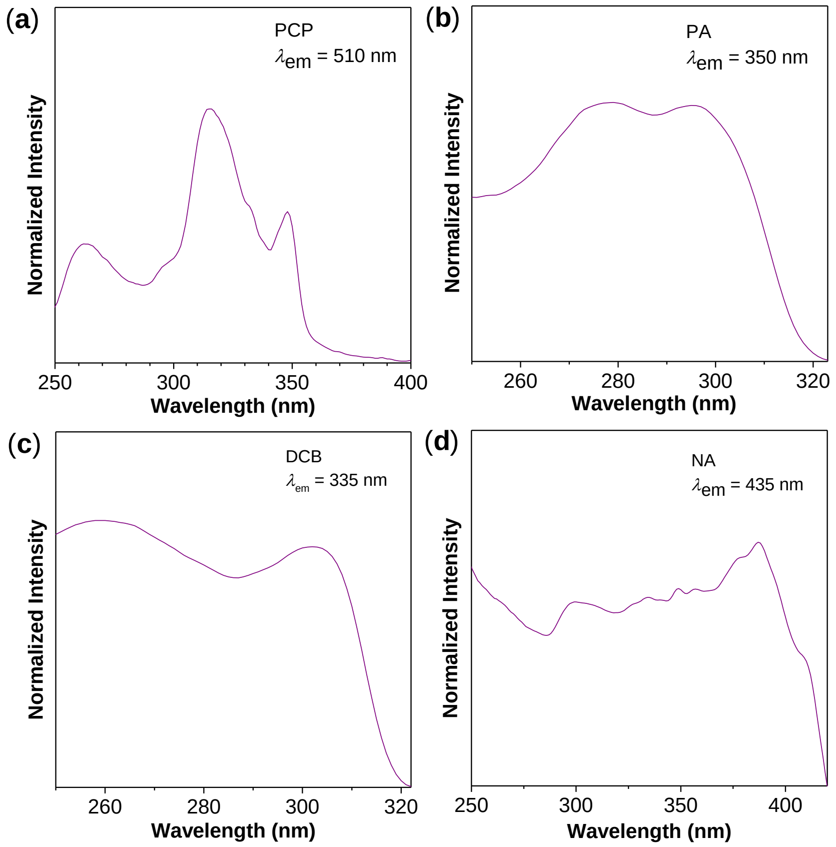

Supplementary Figure 5. Excitation spectra of PCP (a), PA (b), DCB (c) and NA (d) crystals monitored at their emission maximum respectively at 298 K.

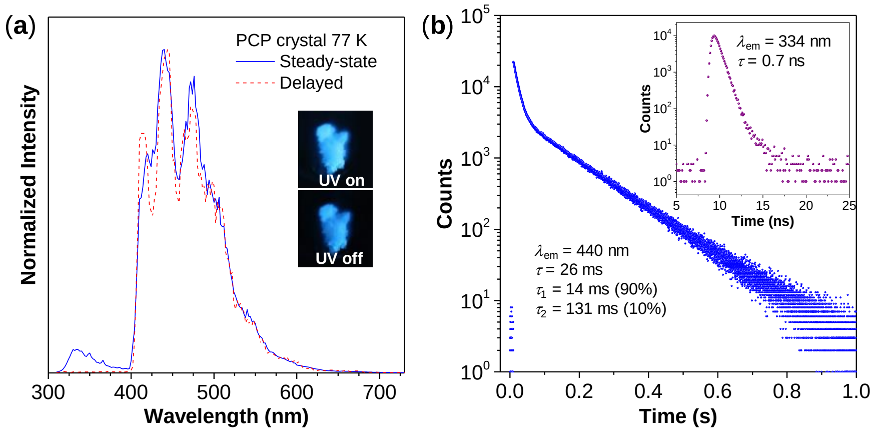

Supplementary Figure 6. (a) Steady-state and delayed (Δt = 50 ms) PL of PCP crystals at 77 K. Insert: Photos showing PCP crystals at 77 K when 254 nm UV light was on and off. (b) Time-resolved fluorescence decay curve at 334 nm (λ_{ex} = 280 nm) and phosphorescence decay curve at 440 nm (λ_{ex} = 254 nm) of PCP crystals at 77 K.

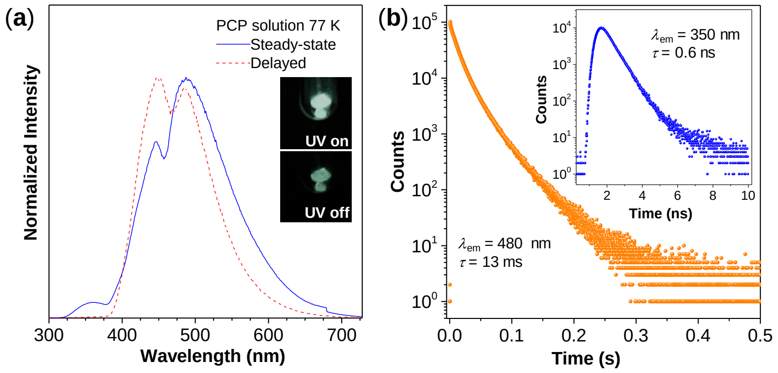

Supplementary Figure 7. (a) Steady-state and delayed (Δt = 50 ms) PL of PCP (10^{-3} M) in EtOH/MeOH (*v*/*v* = 4/1) at 77 K. (b) Time-resolved fluorescence decay curve at 350 nm (λ_{ex} = 280 nm) and phosphorescence decay curve at 440 nm (λ_{ex} = 254 nm) of PCP (10^{-3} M) in EtOH/MeOH (*v*/*v* = 4/1) at 77 K.

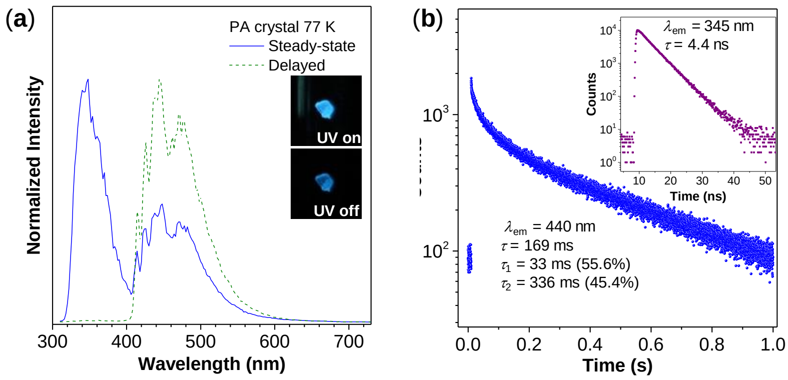

Supplementary Figure 8. (a) Steady-state and delayed emission of PA crystal at 77 K. (b) Time-resolved fluorescence decay curves at 345 nm (λ_{ex} = 280 nm) and phosphorescence decay curve at 440 nm (λ_{ex} = 254 nm) of PA crystals at 77 K.

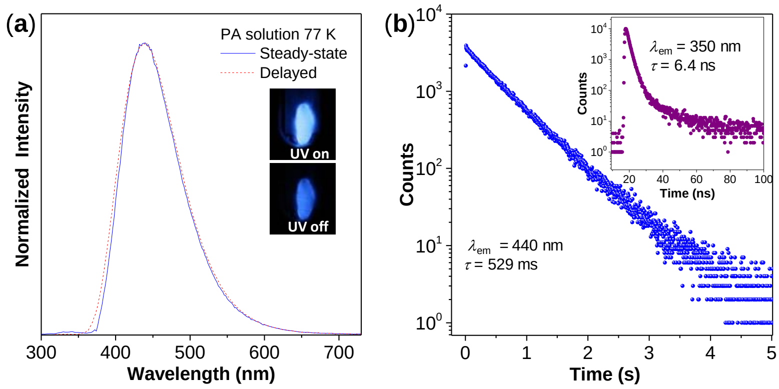

Supplementary Figure 9. (a) Steady-state and delayed PL of PA (10^{-3} M) in EtOH/MeOH (v/v = 4/1) at 77 K. (b) Time-resolved fluorescence decay curve at 350 nm (λ_{ex} = 280 nm) and phosphorescence decay curve at 440 nm (λ_{ex} = 254 nm) of PA (10^{-3} M) in EtOH/MeOH (v/v = 4/1) at 77 K.

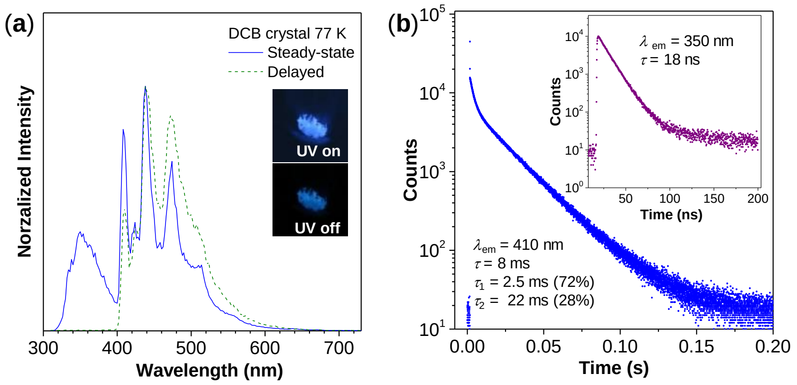

Supplementary Figure 10. (a) Steady-state and delayed PL of DCB crystal at 77 K. (b) Time-resolved fluorescence decay curves at 334 nm (λ_{ex} = 280 nm) and phosphorescence decay curve at 440 nm (λ_{ex} = 254 nm) of DCB crystals at 77 K.

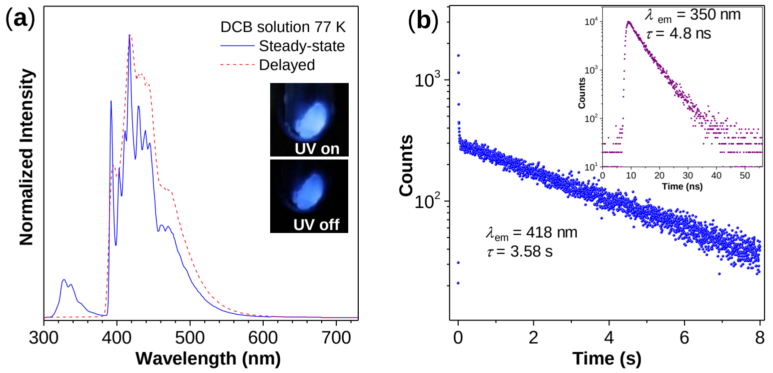

Supplementary Figure 11. (a) Steady-state and delayed PL of DCB (10^{-3} M) in EtOH/MeOH (v/v = 4/1) at 77 K. (b) Time-resolved fluorescence decay curve at 350 nm (λ_{ex} = 280 nm) and phosphorescence decay curve at 420 nm (λ_{ex} = 254 nm) of DCB (10^{-3} M) in EtOH/MeOH (v/v = 4/1) at 77 K.

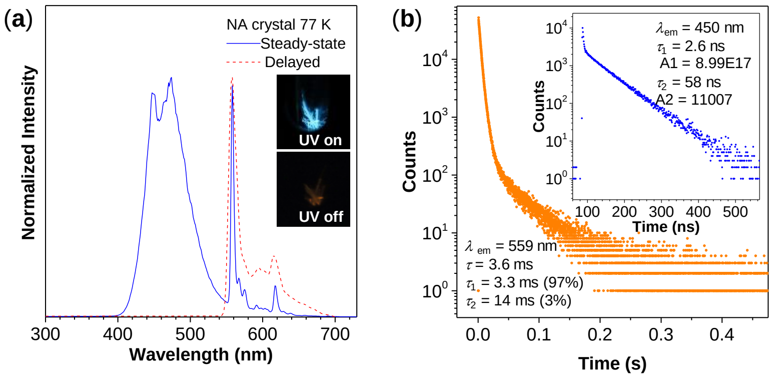

Supplementary Figure 12. (a) Steady-state and delayed PL of NA crystal at 77 K. (b) Time-resolved fluorescence decay curves at 450 nm (λ_{ex} = 280 nm) and phosphorescence decay curve at 559 nm (λ_{ex} = 254 nm) of NA crystals at 77 K.

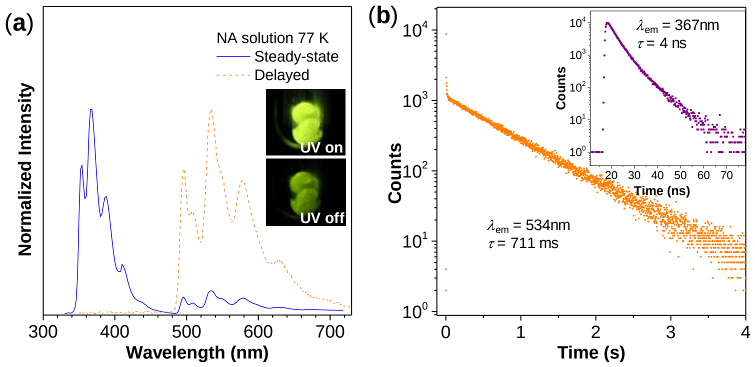

Supplementary Figure 13. (a) Steady-state and delayed PL of NA (10^{-3} M) in EtOH/MeOH (*v*/*v* = 4/1) at 77 K. (b) Time-resolved fluorescence decay curve at 367 nm (λ_{ex} = 280 nm) and phosphorescence decay curve at 440 nm (λ_{ex} = 254 nm) of NA (10^{-3} M) in EtOH/MeOH (*v*/*v* = 4/1) at 77 K.

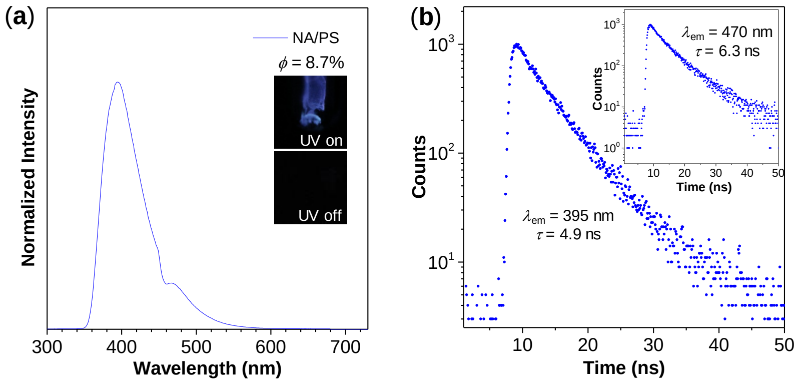

Supplementary Figure 14. (a) Steady-state PL spectrum of NA in PS (m:m = 1:100) under N_2 (λ_{ex} = 254 nm) at RT. (b) Time-resolved PL-decay curve of NA in PS at 395 nm and 470 nm (λ_{ex} = 280 nm) at RT.

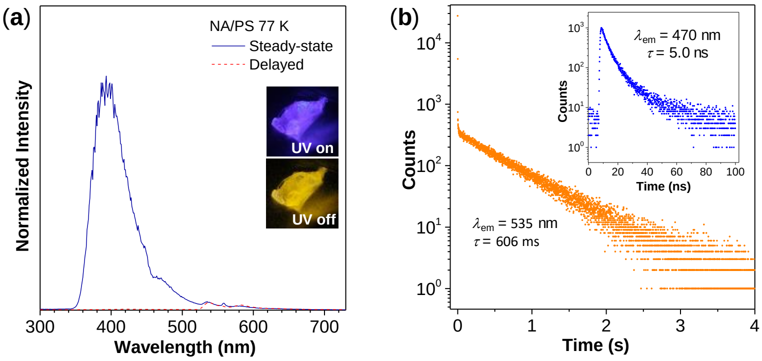

Supplementary Figure 15. (a) Steady-state PL spectrum of NA in PS ((m:m = 1:100, λ_{ex} = 254 nm) at 77 K. (b) Time-resolved PL-decay curve of NA in PS at 395 nm and 470 nm (λ_{ex} = 280 nm) at RT.

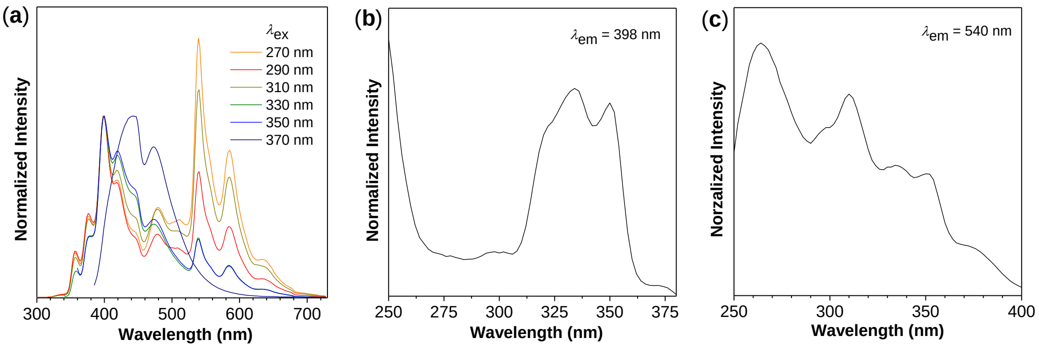

Supplementary Figure 16. (a) Steady-state PL spectra of NA/PCP at RT at various excitation wavelengths. (b) Excitation spectra of NA/PCP at λ_{em}= 398 nm at RT. (c) Excitation spectra of NA/PCP at λ_{em}= 540 nm at RT. NA/PCP was prepared by melt-casting using a starting mass ratio of NA:PCP = 1:100.

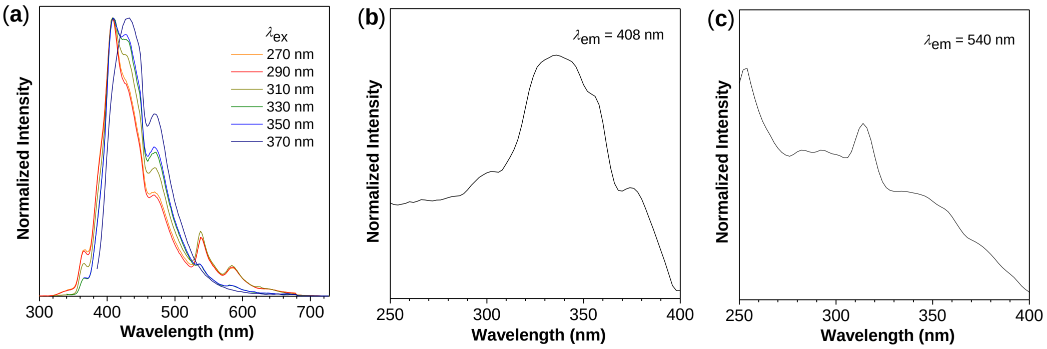

Supplementary Figure 17. (a) Steady-state PL spectra of NA/PA at RT at various excitation wavelengths. (b) Excitation spectra of NA/PA at λ_{em}= 398 nm at RT. (c) Excitation spectra of NA/PA at λ_{em}= 540 nm at RT. NA/PA was prepared by melt-casting using a starting mass ratio of NA:PA = 1:100.

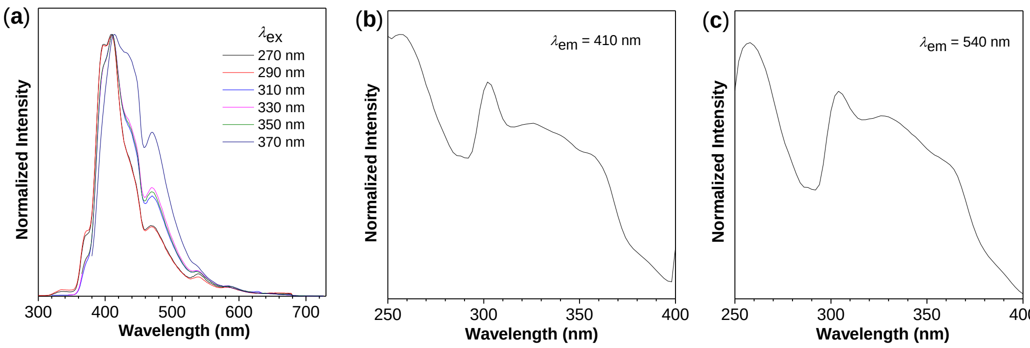

Supplementary Figure 18. (a) Steady-state PL spectra of NA/DCB at RT at various excitation wavelengths. (b) Excitation spectra of NA/DCB at λ_{em}= 410 nm at RT. (c) Excitation spectra of NA/DCB at λ_{em}= 540 nm at RT. NA/DCB was prepared by melt-casting using a starting mass ratio of NA:DCB = 1:100.

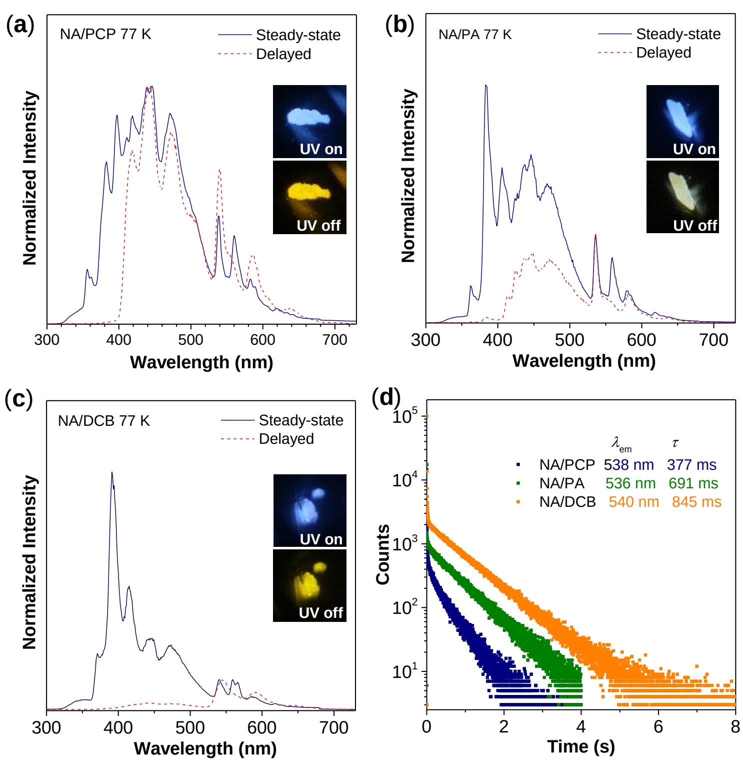

Supplementary Figure 19. (a) Steady-state and delayed PL spectra of NA/PCP (λ_{ex} = 254 nm) at 77 K. Insert: photos of NA/PCP under 254 nm UV light irradiation and after UV irradiation ceased at 77K. (b) and (c) are the same as (a) except that NA/PA and NA/DCB were used respectively instead of NA/PCP. (d) Time-resolved PL-decay curve at 540 nm (λ_{ex} = 254 nm) at 77 K. NA/PCP, NA/PA, NA/DCB were obtained using a melt-casting method with a starting mass ratio of NA:PCP = NA:PA = NA:DCB = 1:100.

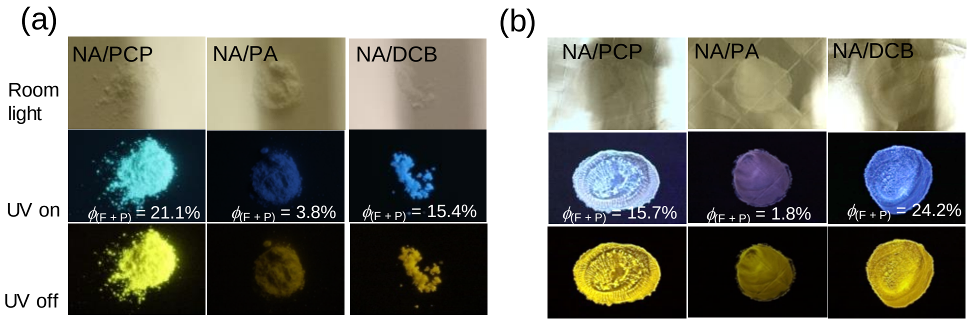

Supplementary Figure 20. (a) Photos of NA/PCP (left), NA/PA (middle), NA/DCB (right) under room light (top), 254 nm UV light irradiation (middle) and after the irradiation ceased (bottom). The samples were obtained by grinding PCP, PA and DCB with NA respectively with a starting mass ratio of NA:PCP = NA:PA = NA:DCB = 1:100. (b) The same as (a) except that NA/PCP, NA/PA, NA/DCB were obtained by drying a drop of EA solution of NA/PCP (*m*/*m* = 1/100) (c_{PCP} = 10 g L^{-1}), NA/PA (*m*/*m* = 1/100) (c_{PA} = 10 g L^{-1}), NA/DCB (*m*/*m* = 1/100) (c_{DCB} = 10 g L^{-1}) respectively on aluminum foil.

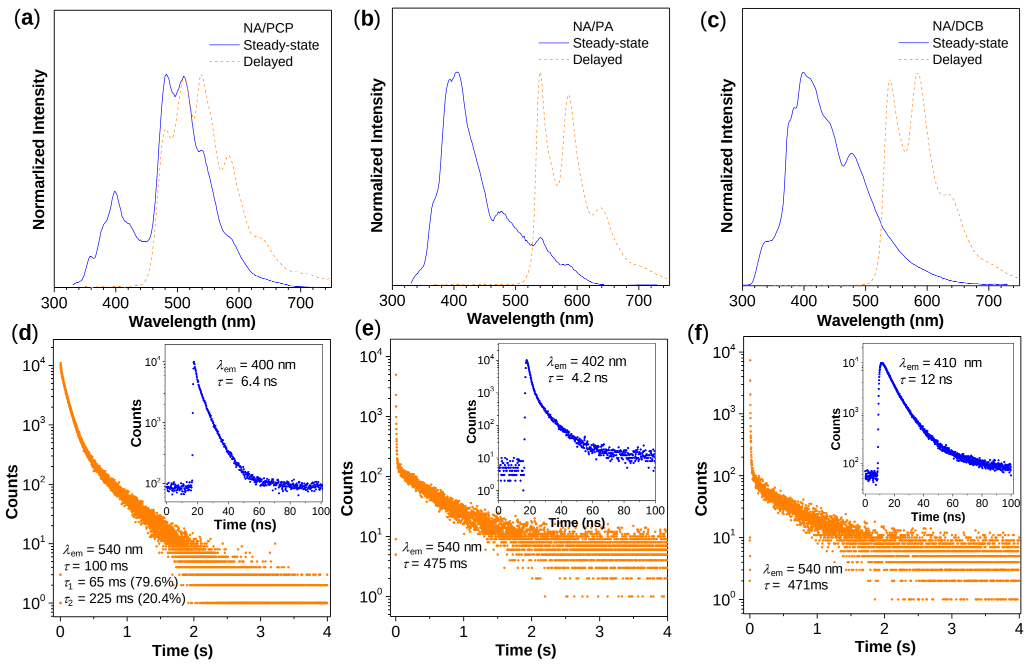

Supplementary Figure 21. Emission spectra and lifetimes of the guest/host systems. (a) Steady-state and delayed (Δt = 50 ms) PL spectra of NA/PCP at ambient conditions. (b) and (c) are the same as (a) except that NA/PA and NA/DCB were used, respectively, instead of NA/PCP. (d) Time-resolved decay curves of NA/PCP at maximum phosphorescence emission (λ_{ex} = 254 nm) and at maximum fluorescence emission (insert, λ_{ex} = 280 nm) at ambient conditions. (e) and (f) are the same as (d) except that NA/PA and NA/DCB were used, respectively, instead of NA/PCP. The samples were obtained by grinding PCP, PA and DCB with NA respectively with a starting mass ratio of NA:PCP = NA:PA = NA:DCB = 1:100.

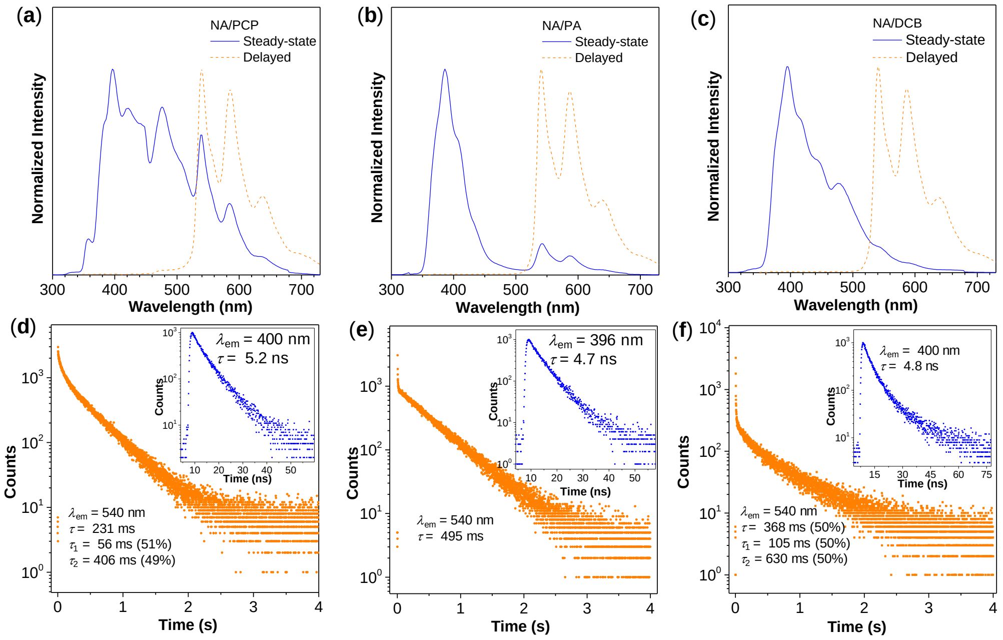

Supplementary Figure 22. Emission spectra and lifetimes of the guest/host systems. (a) Steady-state and delayed (Δt = 50 ms) PL spectra of NA/PCP at ambient conditions. (b) and (c) are the same as (a) except that NA/PA and NA/DCB were used, respectively, instead of NA/PCP. (d) Time-resolved decay curves of NA/PCP at maximum phosphorescence emission (λ_{ex} = 254 nm) and at maximum fluorescence emission (insert, λ_{ex} = 280 nm) at ambient conditions. (e) and (f) are the same as (d) except that NA/PA and NA/DCB were used, respectively, instead of NA/PCP. The samples were obtained by drying a drop of EA solution of NA/PCP (*m*/*m* = 1/100) (c_{PCP} = 10 g L^{-1}), NA/PA (*m*/*m* = 1/100) (c_{PA} = 10 g L^{-1}), NA/DCB (*m*/*m* = 1/100) (c_{DCB} = 10 g L^{-1}) respectively on aluminum foil.

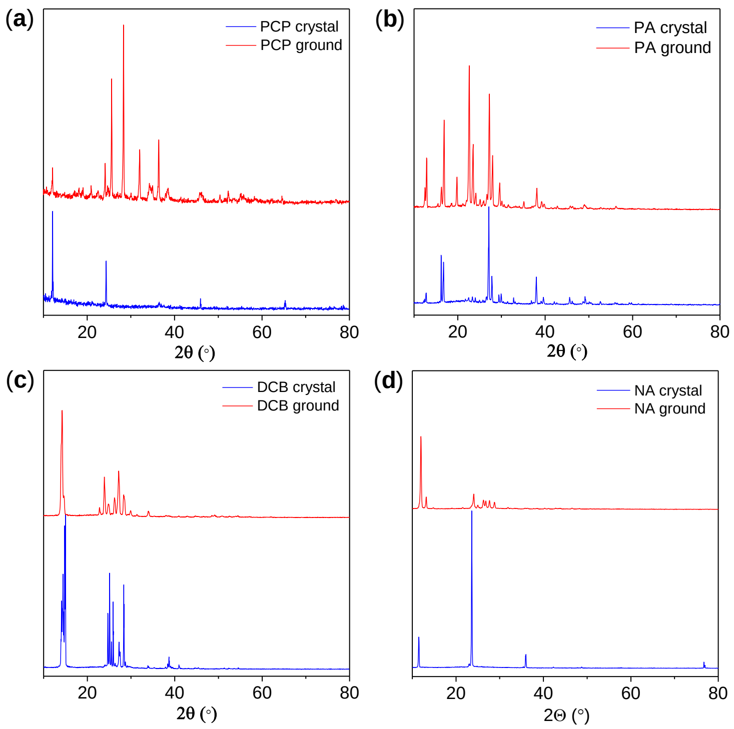

Supplementary Figure 23. Powder XRD patterns of PCP (a), PA (b), DCB (c) and NA (d) crystals before and after grinding.

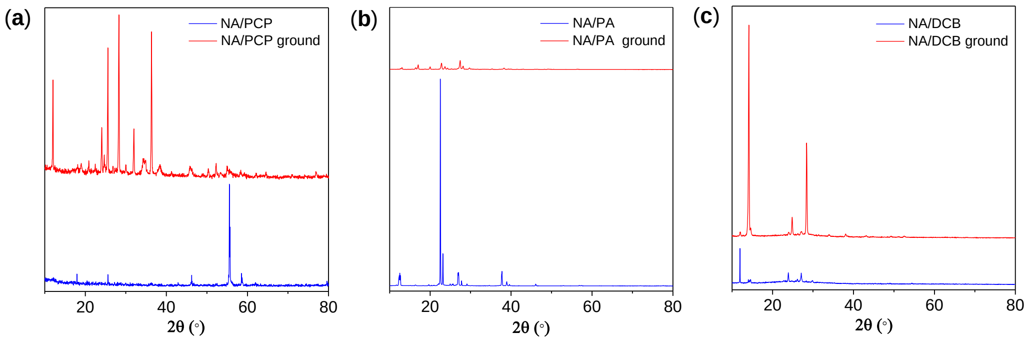

Supplementary Figure 24. Powder XRD patterns of NA/PCP (a), NA/PA (b) and NA/DCB(c) before and after grinding.

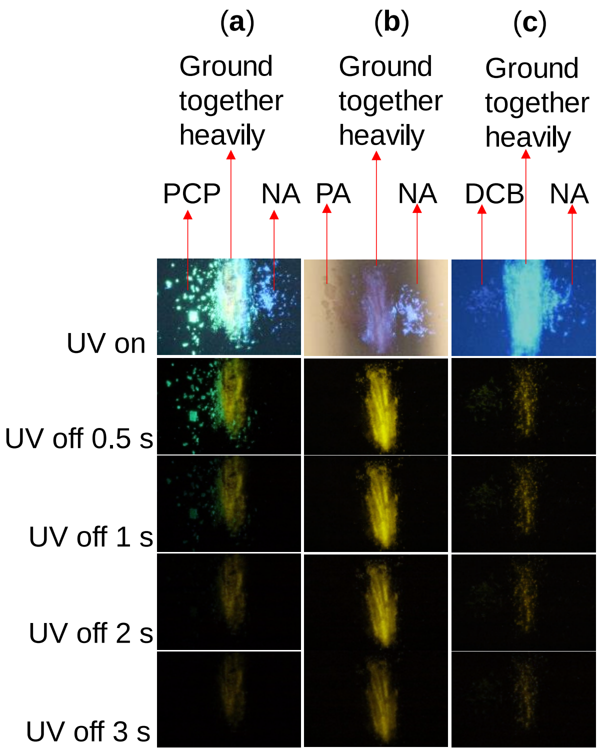

Supplementary Figure 25. (a) Photos of separate PCP, NA crystals and the part where they were smeared together on a filter paper under 254 nm UV light irradiation and after the irradiation ceased. (b) Photos of separate PA, NA crystals and the part where they were smeared together on a filter paper under 254 nm UV light irradiation and after the irradiation ceased. (c) Photos of separate DCB, NA crystals and the part where they were smeared together on a filter paper under 254 nm UV light irradiation and after the irradiation ceased.

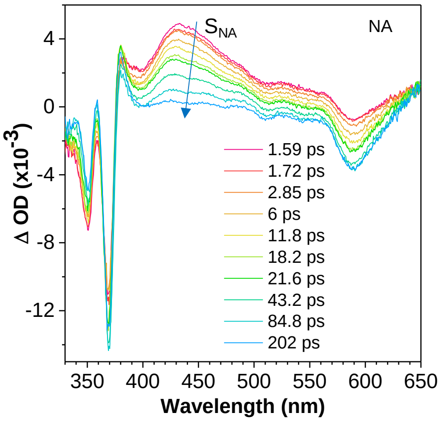

Supplementary Figure 26. Femtosecond transient absorption spectra of NA film. Excitation: 267 nm laser pulse.

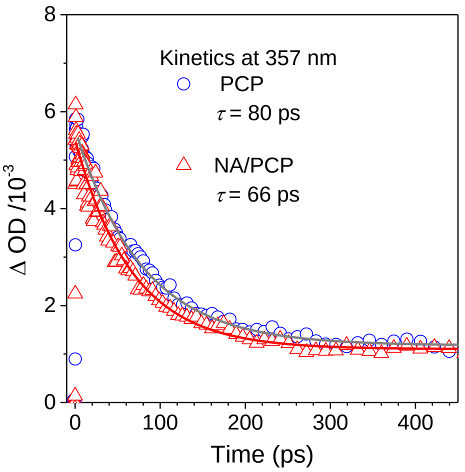

Supplementary Figure 27. Femtosecond transient absorption kinetics of PCP and NA/PCP film at 357 nm. Excitation: 267 nm laser pulse.

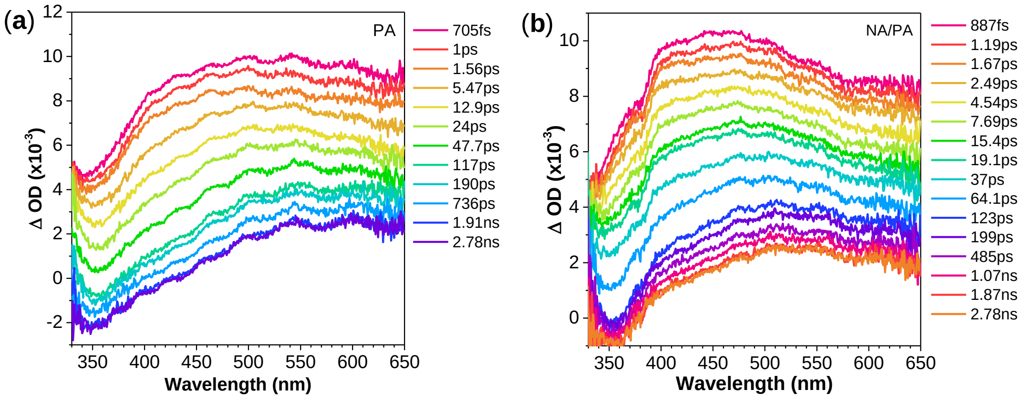

Supplementary Figure 28. Femtosecond transient absorption spectra of PA film (**a**) and NA/PA film (**b**) respectively at different delay times. Excitation: 267 nm laser pulse.

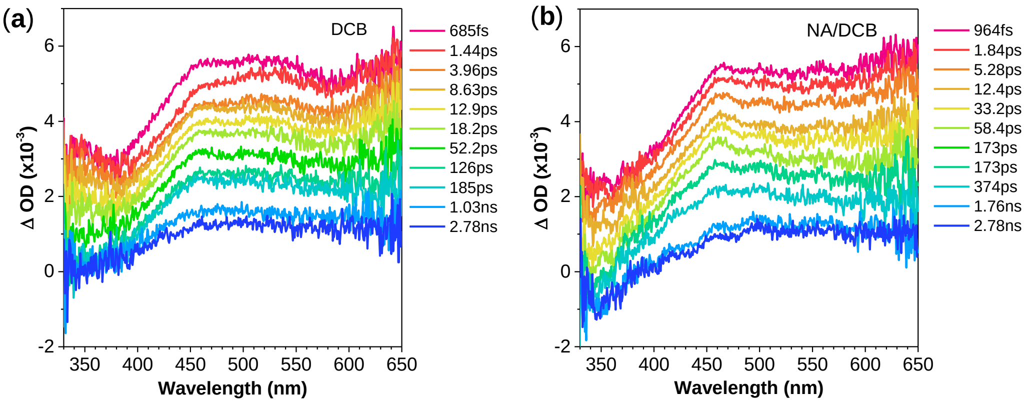

Supplementary Figure 29. Femtosecond transient absorption spectra of DCB film (a) and NA/DCB film (b) respectively at different delay times. Excitation: 267 nm laser pulse.

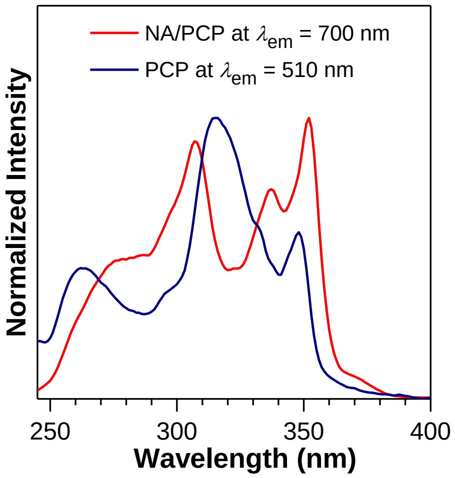

Supplementary Figure 30. Comparison of excitation spectrum of NA/PCP monitored at λ_{em} = 700 nm and that of PCP crystals monitored at λ_{em} = 510 nm at RT.

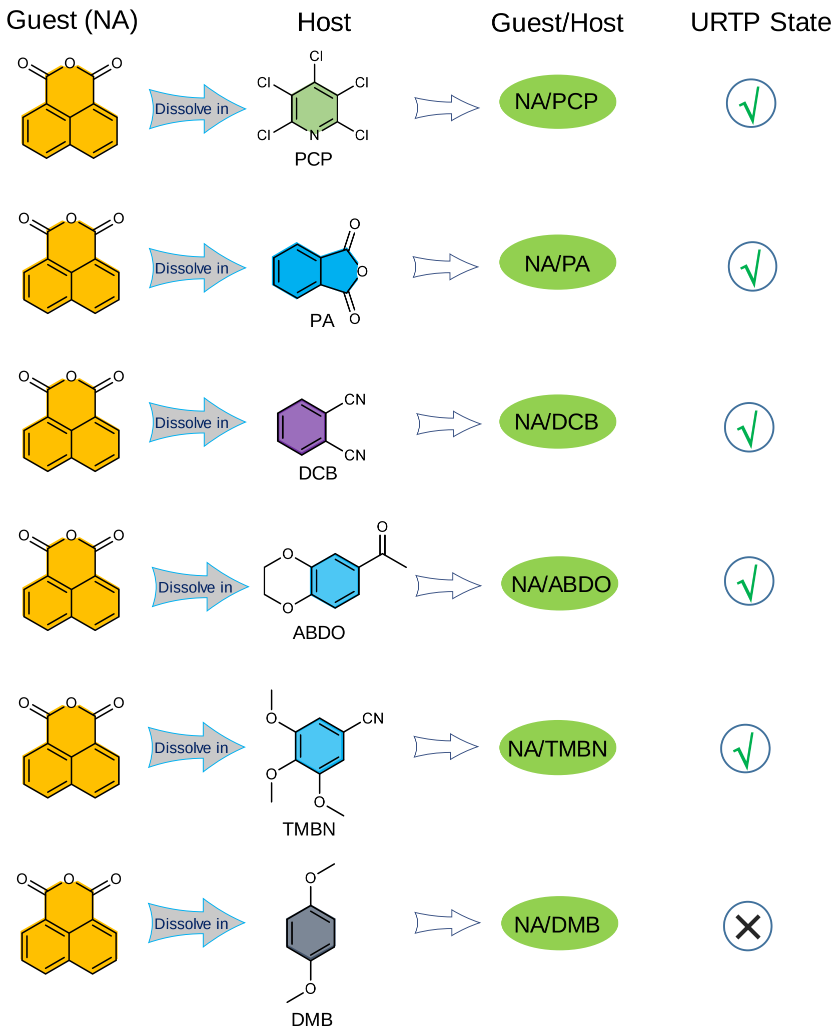

Supplementary Figure 31. Comparison of different host structures and corresponding URTP state of NA/host solid-state soluitons. Abbreviations: ABDO for 6-acetyl-1,4-benzodioxane, TMBN for 3,4,5-trimethoxybenzonitrile, DMB for dimethoxybenzene.

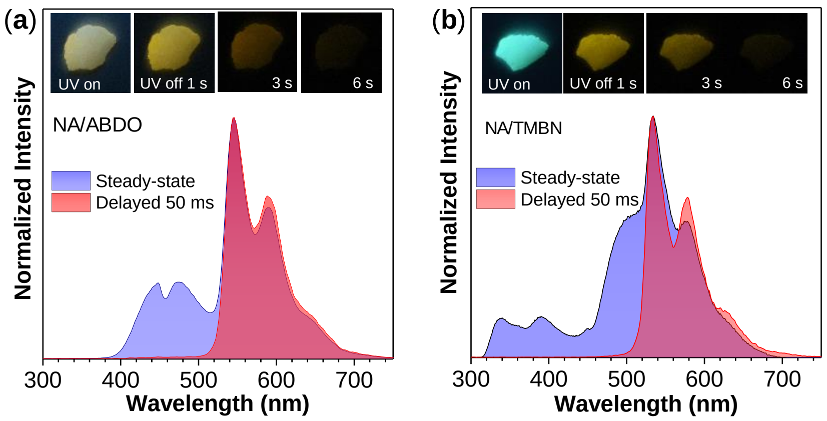

Supplementary Figure 32. (**a**) Steady-state and delayed PL spectra of NA/ABDO (λ_{ex} = 254 nm) at ambient conditions. Insert: photos of NA/ABDO under 254 nm UV light irradiation and after UV irradiation ceased for different times. (**b**) is the same as (**a**) except that NA/TMBN was used instead of NA/ABDO.

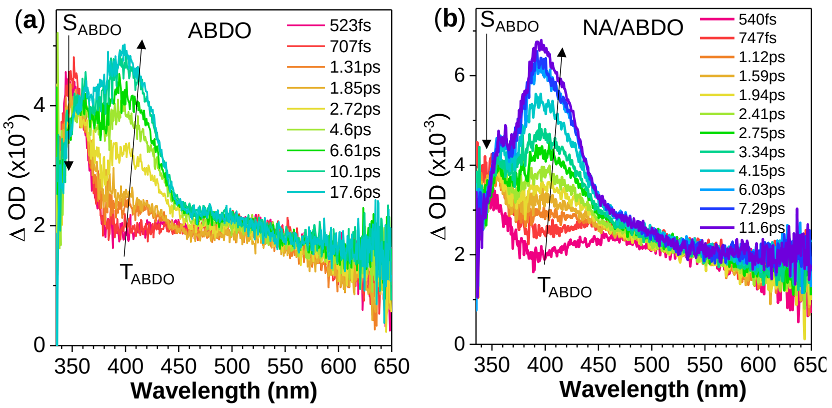

Supplementary Figure 33. Femtosecond transient absorption spectra of ABDO film (**a**) and NA/ABDO film (**b**) respectively at different delay times. Excitation: 267 nm laser pulse.

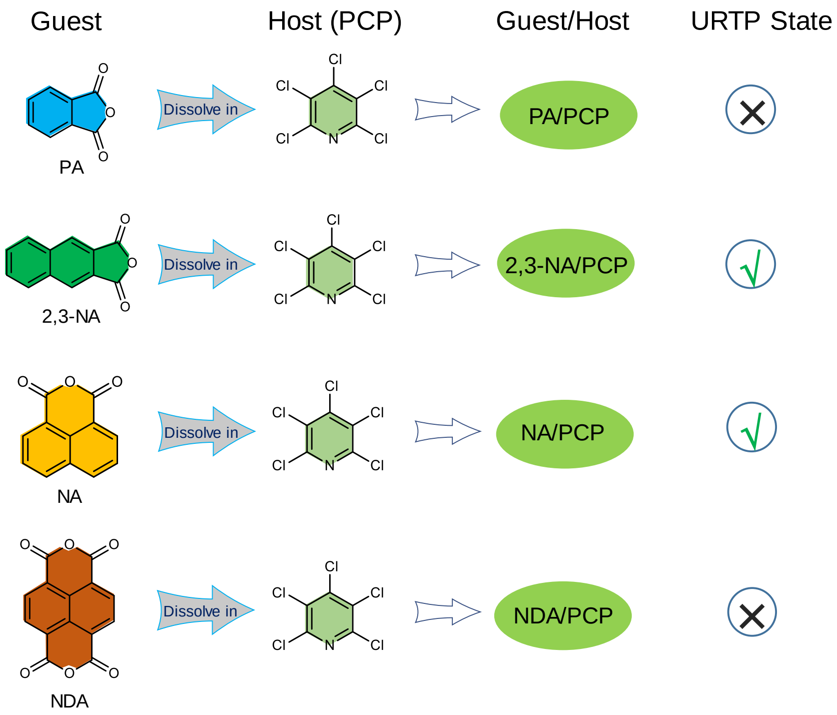

Supplementary Figure 34. Comparison of different guest structures and corresponding URTP state of guest/PCP solid state soluitons. Abbreviations: 2,3-NA for 2,3-naphthalenedicarboxylic anhydride, NDA for 1,4,5,8-naphthalenetetracarboxylic dianhydride.

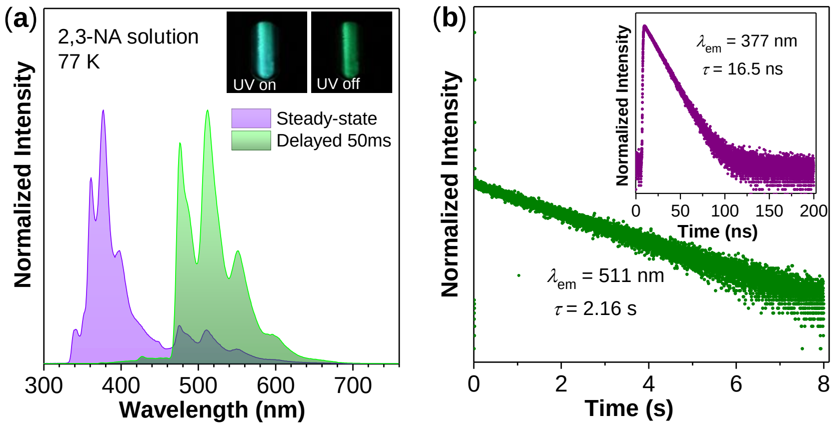

Supplementary Figure 35. (a) Steady-state and delayed PL of 2,3-NA (10^{-3} M) in EtOH/MeOH (*v*/*v* = 4/1) at 77 K (λ_{ex} = 254 nm). (b) Time-resolved fluorescence decay curve at 377 nm (λ_{ex} = 280 nm) and phosphorescence decay curve at 511 nm (λ_{ex} = 254 nm) of 2,3-NA (10^{-3} M) in EtOH/MeOH (*v*/*v* = 4/1) at 77 K.

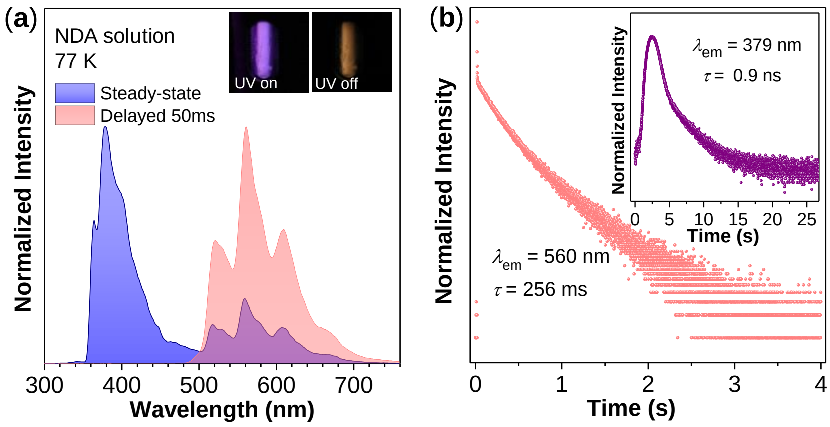

Supplementary Figure 36. (a) Steady-state and delayed PL of NDA (10^{-3} M) in EtOH/MeOH (*v*/*v* = 4/1) at 77 K (λ_{ex} = 254 nm). (b) Time-resolved fluorescence decay curve at 379 nm (λ_{ex} = 280 nm) and phosphorescence decay curve at 560 nm (λ_{ex} = 254 nm) of NDA (10^{-3} M) in EtOH/MeOH (*v*/*v* = 4/1) at 77 K.

Supplementary Table 1. Maximal fluorescence and phosphorescence peaks of PA, 2,3-NA, NA and NDA in rigid glass matrix (MeOH/EtOH, *v*/*v*, 4/1, 10^{-3} M) at 77 K.

	Fluorescence (nm)	Phosphorescence (nm)
PA	350	439
2,3-NA	377	511
NA	367	534
NDA	379	560

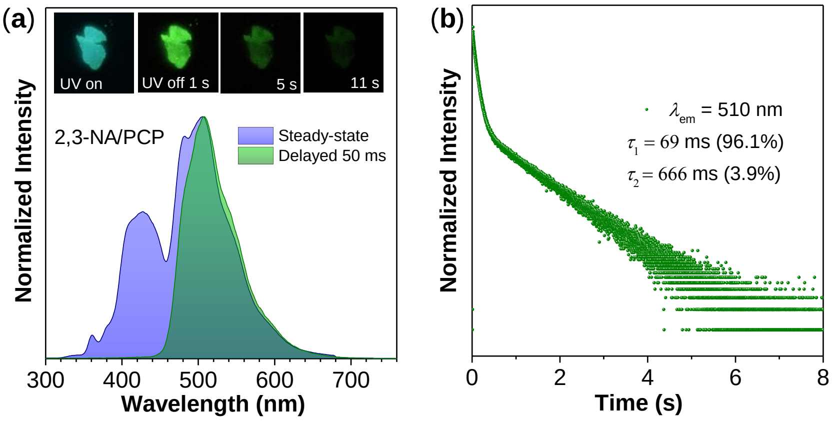

Supplementary Figure 37. (**a**) Steady-state and delayed PL spectra of 2,3-NA/PCP (λ_{ex} = 254 nm) at ambient conditions. Insert: photos of NA/ABDO under 254 nm UV light irradiation and after UV irradiation ceased for different times. (**b**) Time-resolved decay curves of 2,3-NA/PCP at maximum phosphorescence emission (λ_{ex} = 254 nm) at ambient conditions. 2,3-NA/PCP was obtained using a melt-casting method with a starting mass ratio of 2,3-NA:PCP = 1:100.

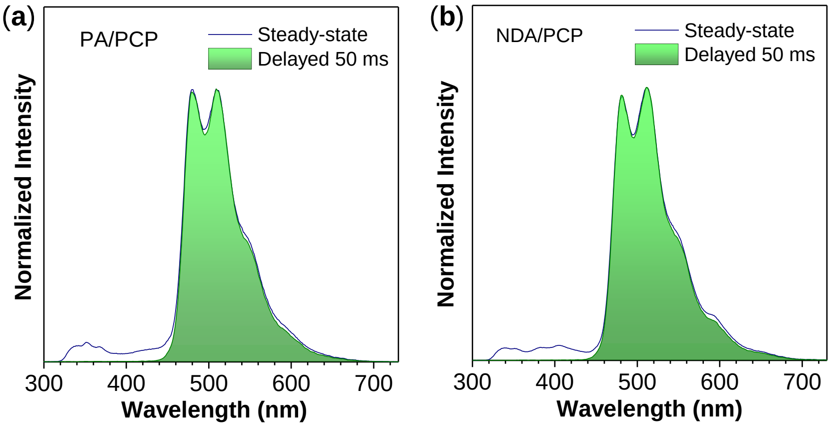

Supplementary Figure 38. Steady-state and delayed PL spectra of PA/PCP (a) and NDA/PCP (b) respectively at ambient conditions (λ_{ex} = 254 nm). PA/PCP and NDA/PCP were obtained using a melt-casting method with a starting mass ratio of PA:PCP = NDA:PCP = 1:100.
