# Supplementary material for: Ultralong UV/mechano-excited room temperature phosphorescence from purely organic cluster excitons
Source: Nat Commun. 2019 Nov 14;10:5161. doi: 10.1038/s41467-019-13048-x (PMC6856348; doi:10.1038/s41467-019-13048-x)
Supplement: Supplementary file 3 — Description of Additional Supplementary Files [file 41467_2019_13048_MOESM3_ESM.pdf]

Description of Additional Supplementary Files

File Name: Supplementary Movie 1
Description: ML of PCP crystals.

File Name: Supplementary Movie 2
Description: ML of DCB crystals.

File Name: Supplementary Movie 3
Description: ML of NA/PCP solid state solution.

File Name: Supplementary Movie 4
Description: ML of NA/PA solid state solution.

File Name: Supplementary Movie 5
Description: ML of NA/DCB solid state solution.
